# A randomized controlled trial on analgesic effect of repeated Quadratus Lumborum block versus continuous epidural analgesia following laparoscopic nephrectomy

**DOI:** 10.1186/s12871-019-0891-7

**Published:** 2019-12-05

**Authors:** Dita Aditianingsih, Pryambodho, Naufal Anasy, Aida Rosita Tantri, Chaidir Arif Mochtar

**Affiliations:** 1 Department of Anesthesiology and Intensive Care, Cipto Mangunkusumo General Hospital, Universitas Indonesia, 6th Salemba Raya, DKI Jakarta, 10430 Indonesia; 2 Department of Urology, Cipto Mangunkusumo General Hospital, Universitas Indonesia, DKI Jakarta, Indonesia

**Keywords:** Epidural analgesia, Laparoscopic nephrectomy, Postoperative analgesia, Patient-controlled analgesia, Quadratus lumborum block

## Abstract

**Background:**

Epidural analgesia as the effective pain management for abdominal surgery has side effects such as paresthesia, hypotension, hematomas, and impaired motoric of lower limbs. The quadratus lumborum block (QLB) has potential as an abdominal truncal block, however, its analgesic efficacy has never been compared to epidural analgesia on laparoscopic nephrectomy. This prospective randomized controlled study compared the effectiveness of QLB with the epidural analgesia technique in relieving postoperative pain following transperitoneal laparoscopic nephrectomy.

**Methods:**

Sixty-two patients underwent laparoscopic donor nephrectomy and were randomized to receive QLB (*n* = 31) or continuous epidural (*n* = 31). The QLB group received bilateral QLB using 0.25% bupivacaine and the epidural group received 6 ml/h of 0.25% bupivacaine for intraoperative analgesia. As postoperative analgesia, the QLB group received repeated bilateral QLB with the same dose and the epidural group received 6 ml/h of 0.125% bupivacaine for 24 h after surgery completion. The primary outcome was the 24-h cumulative morphine requirement after surgery. The secondary outcome was the postoperative pain scores. Sensory block coverage, hemodynamic changes, Bromage score, postoperative nausea-vomiting (PONV), paresthesia, and duration of urinary catheter usage were recorded and analyzed.

**Result:**

The 24-h cumulative morphine requirement and pain scores after surgery were comparable between the QLB and epidural groups. The coverage of QLB was extended from T9 to L2 and the continuous epidural block was extended from T8 to L3 dermatomes. The mean arterial pressure (MAP) measured at 24 h after surgery was lower in the epidural group (*p* = 0.001). Bromage score, incidence of PONV, and paresthesia were not significantly different between the two groups. Duration of urinary catheter usage was shorter (*p* < 0.001) in the QLB group.

**Conclusion:**

The repeated QLB had a similar 24-h cumulative morphine requirement, comparable postoperative pain scores and sensory blockade, higher postoperative MAP, a similar degree of motoric block, no difference in the incidence of PONV and paresthesia*,* and shorter urinary catheter usage, compared to the continuous epidural analgesia following transperitoneal laparoscopic nephrectomy.

**Trial registration:**

ClinicalTrial.gov NCT03520205 retrospectively registered on May 9th 2018.

## Background

The management of postoperative pain holds an important role in laparoscopic living donor nephrectomy patient’s recovery [1, 2]. The postoperative pain after laparoscopic nephrectomy remains a major concern because some patients still demonstrate acute pain that is not different between laparoscopic or open nephrectomy [2, 3]. Epidural analgesia is the gold standard for abdominal surgery including for laparoscopic nephrectomy, however, it has unfavorable side effects such as paresthesia, hypotension, hematomas, impaired motoric of lower limbs and urinary retention that could delay recovery. Administration of intravenous patient-controlled analgesia (PCA) opioid has potential side effects such as sedation, pruritus, nausea, vomiting, and respiratory depression [4].

Blanco and colleagues first introduced the quadratus lumborum block (QLB) by injecting local anesthetic in the interfascial plane at the anterolateral margin of quadratus lumborum muscle (the lateral QLB) under ultrasound guidance and then modified the point of injection into the posterior wall of quadratus lumborum muscle (the posterior QLB) for postoperative analgesia after cesarean delivery [5]. Børglum and colleagues refined the technique, by adopting the posterior approach of QLB and using the ‘Shamrock’ sign to place the needle tip anterior to the quadratus lumborum muscle, then injecting the local anesthetic agent into the plane between the quadratus lumborum and psoas muscles (the anterior QLB) [6].

Clinical trials of QLB are still limited, but evidence has shown the efficacy of QLB in reducing opioid requirement and postoperative pain to promote recovery after cesarean section, laparoscopic or laparotomy procedure, and hip surgery [5, 7–10]. Transperitoneal laparoscopic donor nephrectomy patients reveal the significant postoperative pain related to the Pfannenstiel incision, port sites wounds, and deep intra-abdominal nociception [2, 3]. For the abdominal surgery, QLB became an alternative to epidural without central neuraxial block side effects [5, 11], however, the efficacy of QLB as perioperative pain management in transperitoneal laparoscopic nephrectomy is unclear. We hypothesized that repeated QLB could be superior to a continuous epidural block in relieving postoperative pain with fewer side effects in living donor patients. Our study compared the effectiveness of QLB to the continuous epidural analgesia in relieving postoperative pain following transperitoneal laparoscopic nephrectomy. The primary outcome was the 24-h cumulative morphine requirement after surgery. The secondary outcome was the 24-h postoperative pain scores. The sensory block coverage, hemodynamic changes, Bromage score, postoperative nausea-vomiting (PONV) occurrence, and duration of urinary catheter usage were recorded during observation.

## Methods

### Patients and study design

This prospective open-label randomized controlled study adheres to CONSORT guideline. This study was approved by the Ethics and Research Committee of Universitas Indonesia (0211/UN2.F1/ETIK/2018) and was retrospectively registered in ClinicalTrials.gov (NCT03520205) on May 9th, 2018 with predicted subject recruitment time from May 1st – October 30th, 2018. Written informed consents were obtained from all adult patients with American Society of Anesthesiologists (ASA) I–II who underwent laparoscopic living donor nephrectomy in Cipto Mangunkusumo Hospital, DKI Jakarta. The inclusion criteria included age 18–60 years with body mass index (BMI) < 30 kg/m^2^. The exclusion criteria included the inability to communicate, history of allergy or contraindication to local anesthetics, contraindication to epidural or QLB (coagulopathy or infections on the injection site).

The research protocol was explained to the patient who met the inclusion criteria. All patients were educated about QLB and continuous epidural procedures, how to describe the degree of pain with the numerical rating scale (NRS), and how to use the PCA morphine pump when the pain level (NRS) ≥ 4 after the surgery. After obtaining written approval, the patients were randomized either into the QLB group (intervention group) or the epidural group (control group). Randomization was conducted in a block size of 4 using a computerized randomization sequence by independent research assistants. The randomization allocation number for each subject was written on paper and put in a closed envelope. The envelope was opened by the anesthesiologist who was appointed to perform epidural or QLB for this study. Primary investigators and statisticians were blinded to data collection throughout the study.

### General management

Standard monitoring was placed such as electrocardiogram, oxygen saturation, non-invasive blood pressure monitors and cardiometry ICON™. General anesthesia induction was conducted with the administration of fentanyl 2 μg/kg as co-induction, proceeded with propofol 1–2 mg/kg. Endotracheal tube intubation was facilitated with atracurium 0.5 mg/kg. The ventilator was set to volume control with positive end-expiratory pressure 5 cmH_2_O and FiO_2_ 30–50%, breathing frequency was adjusted with the end-tidal carbon dioxide target of 35–45 mmHg. The anesthesia was maintained using sevoflurane with 0.8–1.3 minimum alveolar concentration, fresh gas flow 1 L/minute with oxygen compressed air ratio of 40:60, atracurium 0.5 mg/kg/h, to achieve bispectral index of 40–60, end-tidal fraction of sevoflurane 1.6–2.2%, and train of four ratios ≤25% during surgery. All subjects received fentanyl 1 μg/kg i.v. if there was an increase of change in systolic blood pressure or pulse rate > 20% from the initial value during surgery and the total intraoperative fentanyl usage was recorded. Ephedrine was given if there is a decrease in mean arterial pressure (MAP) less than 65 mmHg during the study observation.

All patients were planned for the transperitoneal left laparoscopic nephrectomy and kidney extraction through a Pfannenstiel incision, that was performed by the surgeon (CAM, IW, ARH) who had more than 5 years of experience to performed transperitoneal laparoscopic live donor nephrectomy. At the end of surgery, neostigmine 0.04–0.07 μg/kg was given to reverse residual neuromuscular block and the patient was extubated when had reached train of four ratio of 0.9–1.0.

### Technique of epidural analgesia

Two experienced anesthetist consultants (P, DA) performed all the thoracic epidural and QLB procedures. Patients in the epidural group had an epidural catheter placement procedure in the left lateral decubitus position after intubation under general anesthesia. After ensuring skin asepsis and draping the area with a sterile cover, an 18G Tuohy epidural needle was inserted in vertebral interspace T10–11 and catheter was advanced 5 cm length within the epidural space [12]. We observed vacuum epidural catheter aspiration and a test dose of 3 ml 0.25% bupivacaine with adrenaline 1:200,000 without any change in pulse rate or blood pressure to confirm the position of catheter within the epidural space. Then a continuous epidural infusion of 6 ml/h 0.25% bupivacaine was maintained for intraoperative analgesia. For 24 h postoperative pain management, after completion of the surgery, the continuous epidural dosage was decreased into 0.125% bupivacaine 6 ml/h at the start and the dose was increased in 2 ml/h increments up to 10 ml/h if the pain is still uncontrolled on PCA morphine setting.

### Technique of Quadratus Lumborum block

All patients in the QLB group received the first bilateral ultrasound-guided transmuscular QLB (anterior QLB or QLB3) after induction and intubation under general anesthesia for intraoperative analgesia. Patients were in supine position with the site to be blocked slightly facing upward by pillow underneath it and the tilted operating table. After ensuring skin asepsis of the area, a 2.0–5.5 MHz convex transducer (C5-1E, DC-70, Mindray, Shenzen China) covered with sterile drapes attached in the transverse plane to the inferior lumbar (Petit’s) triangle that consisted of iliac crest in the inferior edge, the latissimus dorsi muscle in the posterior side and external abdominal oblique muscle in the anterior region. The transducer was advanced posteriorly until the Shamrock sign (the erector spinae, quadratus lumborum, and psoas muscles being the leaves, and the transverse process of L4 as the stem) appeared on the ultrasound. A 21G 100-mm peripheral block needle (Stimuplex®, BBraun, Mesulngen Germany) was inserted in-plane with ultrasound probe passing in anterior to posterior direction through the QL muscle and reached the border between the QL and psoas major muscle [6, 11]. After confirming negative for blood aspiration, 1 ml normal saline was injected to obtain hydrodissection sign for verifying the needle tip, then a volume of 0.3–0.4 ml/kg 0.25% bupivacaine with a maximum volume of 25 ml was injected on each side (See Additional file 1). For 24 h postoperative analgesia, the second bilateral QLB procedures were repeated one time at the end of surgery using a similar regimen of 0.3–0.4 ml/kg 0.25% bupivacaine with a maximum volume of 25 ml on each side. (See Additional file 1).

### Postoperative patient-controlled morphine

In the recovery room, patient-controlled morphine i.v. using a portable programmed pump (Perfusor® Space PCA Infusion Pump System, BBraun, Germany) was connected after the patient was fully awake and able to follow the instruction. All patients were informed first at preoperative anesthesia visit and once again at the recovery room after the surgery, how to press the PCA button if they experienced moderate to severe pain. The PCA setting was 1 mg bolus, lockout time 10 min and maximum dosage 6 mg/h, without basal opioid infusion. Ondansetron 4 mg i.v. and omeprazole 40 mg i.v. were administered every 12 h to prevent PONV.

### Study parameters and statistical analysis

This study aimed to compare the analgesic efficacy of QLB to continuous epidural analgesia as postoperative pain management. The primary outcome was the cumulative morphine requirement in 24 h after surgery. Time to first morphine requirement was also recorded. The secondary outcomes were pain scores at rest and movement, and motoric block at time points 2, 6, 12 and 24 h after anesthesia recovery. Pain scores were assessed using NRS (0 = no pain; 1–3 = mild pain, 4–6 = moderate pain, 7–10 = severe pain). Sensory block was assessed after anesthesia recovery using pinprick and cold loss sensation with ice and alcohol. The motoric block was evaluated using Bromage score (grade 1 = free movement of leg and feet; 2 = just able to flex knees with free movement of the feet; 3 = unable to flex knees, free movement of feet; 4 = unable to move the legs or feet). The MAP, pulse rate, and cardiometry cardiac index were recorded at baseline, during surgery, and 24 h after anesthesia recovery. The occurrence of PONV and paresthesia within 24 h postoperatively and urinary catheterization duration were recorded. All of the outcomes were documented by the acute pain service team and ward nurses.

Sample size calculation was based on the primary hypothesis that opioid requirement in 24 h after surgery. Based on the previous study, the mean (standard deviation (SD)) cumulative morphine requirement 24 h after surgery was 4 mg (SD = 4.723) [13]. From a preliminary study, the mean (SD) cumulative morphine requirement 24 h after surgery was 4 mg (SD = 6.09). A sample size of 28 patients in each group was determined with a statistical power of 0.8 and type-1 error of 0.05. This study recruited 62 patients to allow 10% dropouts.

All statistical studies were analyzed using Statistical Package for the Social Sciences version 20 (IBM Corp. 2011, Armonk, NY). Differences between numerical variables were analyzed using the unpaired t-test for normal distribution data and Mann-Whitney test for abnormal distribution data. Differences between categorical variables were analyzed using Chi square test. Numerical data with normal distribution was displayed in a mean (SD), abnormal distribution was displayed in the median (95% confidence interval (CI)) values, and as a percentage for categorical variables. The analysis was statistically significant if the *p*-value was less than 0.05.

## Results

**Fig. 1:**
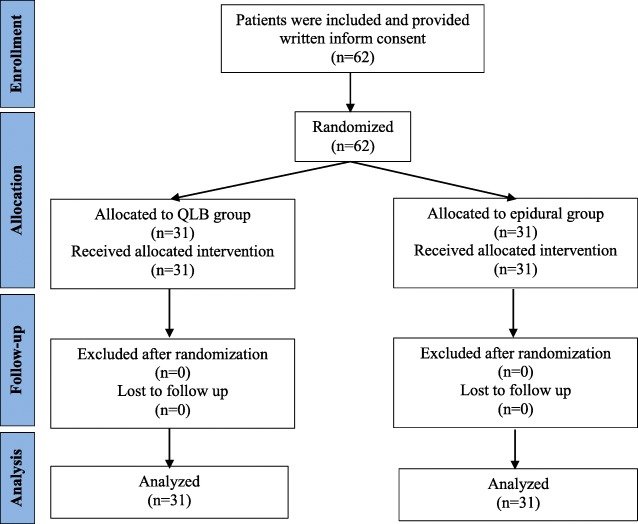
CONSORT flow diagram

We enrolled 62 patients who met the inclusion criteria and signed the informed consent to take part in the study from May 1st – October 30th, 2018. The subjects were randomly assigned into two groups and received their allocated intervention. All patients underwent transperitoneal left laparoscopic nephrectomy and had a Pfannenstiel incision. None of the subjects had subcostal incision or the conversion from laparoscopic to open nephrectomy*.* None of the subjects were excluded from the study; therefore, all patients were followed-up and included in the final analysis (Fig. 1).

Forty-one (66%) subjects are male. Between the QLB and epidural group, the demographic baseline and perioperative characteristics of the study subjects were similar. The intraoperative fentanyl requirement was not significantly different between the two groups. However, ephedrine requirement was higher in the epidural group compared to QLB group (0 (0–50) vs 10 (0–50) mg, *p* = 0.026) (Table 1). The mean total dosage of bupivacaine used in the QLB group was significantly lower than the epidural group (Table 1).

**Table 1:** Patient baseline and perioperative characteristics

Variables	QLB group (*n* = 31)	Epidural group (*n* = 31)	*p* value
Men (%)	21 (67.74)	20 (64.51)	1.000
Age (years)	38.29 ± 12.97	39.97 ± 11.49	0.447
Body weight (kg)	64.34 ± 10.10	62.72 ± 10.06	0.530
Body height (cm)	164.06 ± 7.62	161.80 ± 8.74	0.277
BMI (kg/m^2^)	24.06 ± 3.82	24.13 ± 4.06	0.940
ASA			0.794
ASA 1 (%)	18 (58.06)	20 (64.51)	
ASA 2 (%)	13 (41.94)	11 (35.49)	
Intraoperative fentanyl dosage (μg)	50 (34.66–83.08)	50 (40.73–101.21)	0.442
Intraoperative ephedrine dosage (mg)	0 (1.37–9.59)	10 (5.48–14.39)	0.026
Duration of surgery (minute)	285.6 ± 42.6	292.2 ± 44.4	0.551
Duration of anesthesia (minute)	354.6 ± 36.6	357.6 ± 33.6	0.796
Total bupivacaine dosage (mg)	200.00 ± 0.00	253.19 ± 11.12	< 0.001

*QLB* Quadratus lumborum block, *BMI* Body mass index, *ASA* American Society of Anesthesiologist. Data are presented in number (percentage), mean ± standard deviation or median (95% confidence interval).

**Fig. 2:**
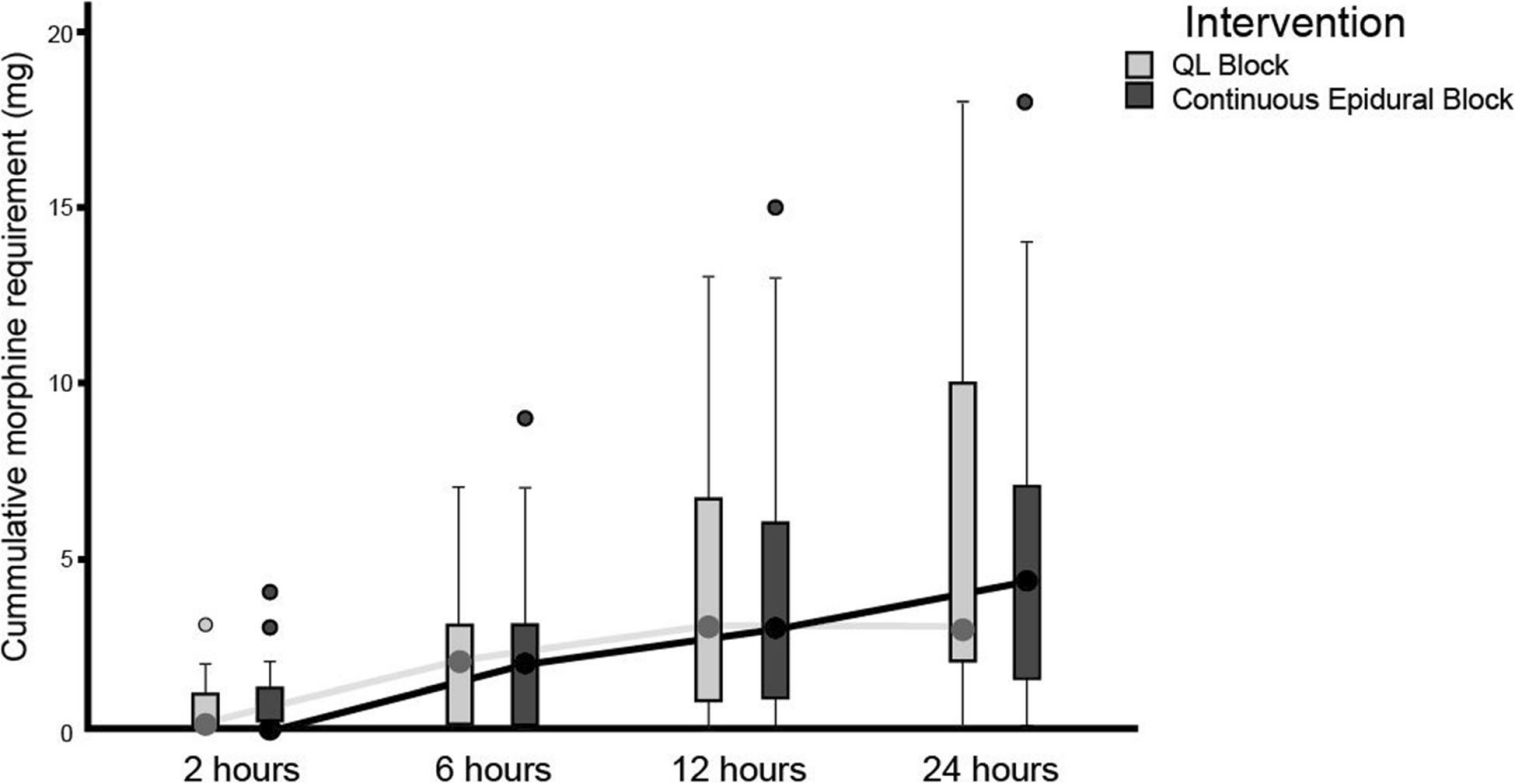
Cumulative morphine requirement of QLB versus continuous epidural analgesia**.** Median (95% CI) values of cumulative morphine requirement (mg) after anesthesia recovery at each time point are as follows: 2 h, 0 (0.34–1.05) vs 0 (0.28–1.10) (*p* = 0.857); 6 h, 2 (0.69–2.08) vs 2 (0.68–1.93) (*p* = 0.977); 12 h, 3 (1.11–2.74) vs 3 (0.97–2.49) (*p* = 0.764); 24 h, 3 (0.69–2.62) vs 4 (0.66–1.88) (*p* = 0.792). The *p*-values were analyzed using Mann-Whitney test, the horizontal lines indicate medians; boxes indicate interquartile range; whiskers indicate range

Figure 2 shows the 24-h cumulative morphine requirement at each time point after surgery were comparable between the QLB and epidural group. Time to first morphine initiation using PCA was not significantly different between the QLB and epidural groups (120 (95% CI: 137.61–354.45) vs 125 (95% CI: 133.56–370.31) minutes, *p* = 0.703) (See Additional file 2). There were 3 subjects in the QLB group and 4 subjects in the epidural group did not need additional morphine at all in 24 h after anesthesia recovery.

Pain scores at rest and in movement at each time point in 24 h after anesthesia recovery were not significantly different between the QLB and epidural groups (Table 2). The lowest pain scores at rest and in movement were at 2 and 6 h after anesthesia recovery in both groups.

**Table 2:** Postoperative pain scores of QLB versus continuous epidural analgesia

Parameter	QLB group (*n* = 31)	Epidural group (*n* = 31)	*p-*value	Mean difference (95% CI)
NRS at rest after anesthesia recovery				
Immediately	1 (1.37–2.78)	2 (1.74–3.34)	0.377	− 0.46 (− 1.50–0.58)
At 2 h	2 (1.44–2.56)	2 (1.48–2.83)	0.719	−0.15 (− 1.01–0.70)
At 6 h	1 (1.38–2.47)	2 (1.53–2.54)	0.751	−0.12 (− 0.84–0.61)
At 12 h	2 (1.54–2.69)	2 (1.59–2.56)	0.916	0.04 (−0.69–0.77)
At 24 h	2 (1.59–2.56)	2 (1.60–2.71)	0.831	−0.08 (− 0.80–0.64)
NRS in movement after anesthesia recovery				
Immediately	3 (2.48–3.98)	3 (2.58–4.18)	0.774	−0.15 (− 1.23–0.92)
At 2 h	3 (2.62–3.84)	3 (2.48–3.83)	0.862	0.08 (−0.81–0.96)
At 6 h	3 (2.59–3.64)	3 (2.62–3.13)	0.916	−0.04 (− 0.77–0.69)
At 12 h	3 (2.90–4.10)	3 (2.85–3.84)	0.685	0.15 (−0.60–0.91)
At 24 h	3 (2.84–3.78)	3 (2.68–3.78)	0.827	0.08 (−0.63–0.78)

*QLB* Quadratus lumborum block, *NRS* Numerical rating scale. Data are presented as mean or median (95% confidence interval). Numerical values are compared using unpaired t-test or and Mann-Whitney test. *P*-values of 0.05 or less are considered significant.

**Fig. 3:**
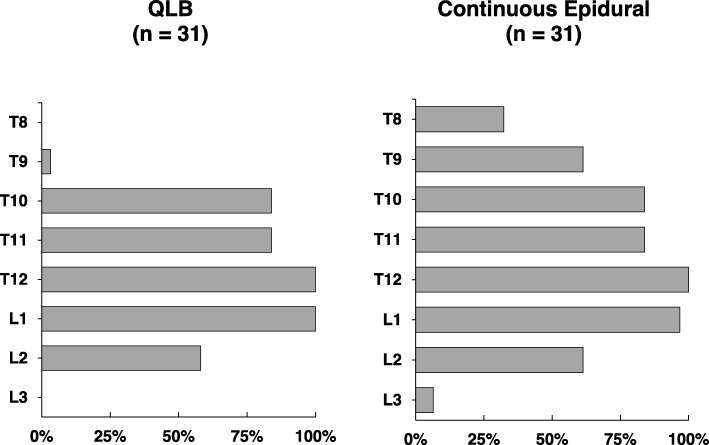
Dermatomal effects of QLB and continuous epidural analgesia**.** The QLB and epidural group showed similar percentage of sensory blockade on level T12–L1 (97–100%), T10–T11 (84%), L2 (58–61% of patients). The QLB group showed percentage of sensory blockade on level T9 (3% vs 61.3%), T8 (0% vs 32.3%), L3 (0% vs 6.5%) less than epidural group patients. QLB, quadratus lumborum block

The bar in Fig. 3 shows the variable degree of sensory block spread in both groups. The majority of patients in the QLB group had the loss of cold and pinprick sensations from T10 to L2, compared with the majority of patients the epidural group had the loss of cold and pinprick sensations from T9 to L2. None of patients in the QLB group showed sensory blockade on level T8 and L3.

**Fig. 4:**
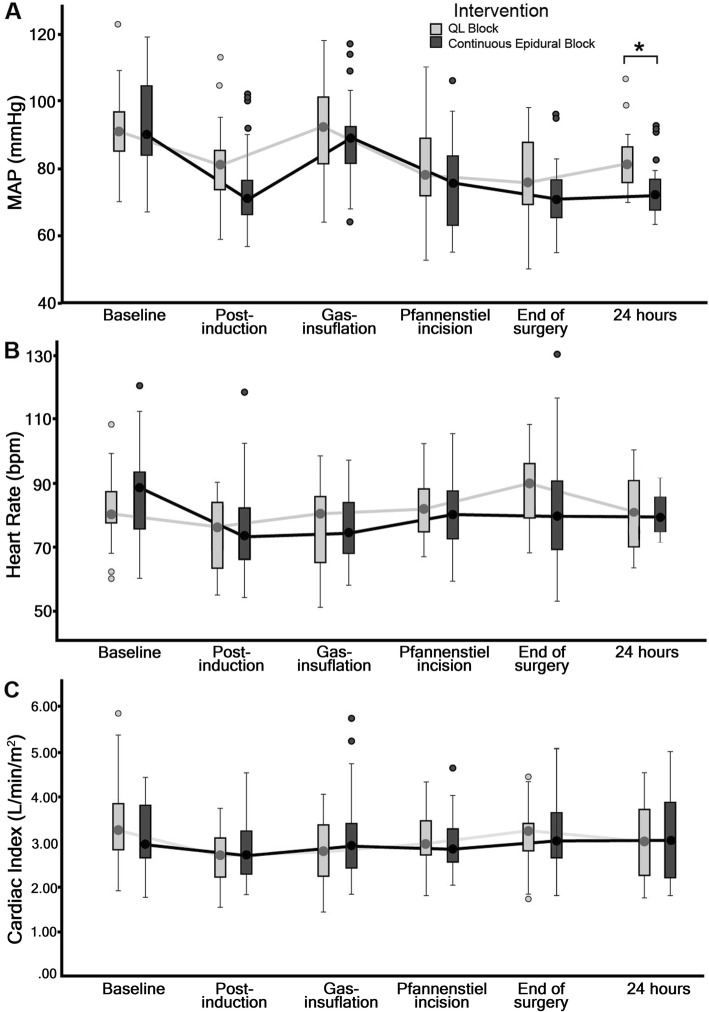
Intraoperative hemodynamic profile of QLB versus continuous epidural analgesia**.** The horizontal lines indicate medians; boxes indicate interquartile range; whiskers indicate range. Median (95%CI) values of hemodynamic parameters at time points as follows: MAP: baseline 91 (87.34–95.76) to 90 (88.36–98.99) (*p* = 0.524); post-induction, 81 (75.76–85.15) to 71 (70.36–79.06) (*p* = 0.072); gas-insufflation 92 (86.84–97.48) to 88 (82.90–92.52) (*p* = 0.210); pfannenstiel incision 78 (75.60–84.47) to 75 (73.91–82.03) (*p* = 0.486); end of surgery 75 (72.48–81.32) to 71 (68.22–75.17) (*p* = 0.063); 24 h 83.33 (78.72–87.95) to 72.26 (67.69–76.83) (*p* = 0.001); HR: baseline 80 (77.67–85.30) to 88 (80.16–90.93) (*p* = 0.215); post-induction 76 (70.87–78.10) to 73 (70.00–80.45) (*p* = 0.816); gas-insufflation 80 (71.48–81.16) to 74 (72.27–79.80) (*p* = 0.855); pfannenstiel incision 81 (77.95–90.89) to 80 (76.75–84.73) (*p* = 0.447); end of surgery 90 (83.64–92.17) to 80 (75.14–88.02) (*p* = 0.049); 24 h, 82 (64–100) to 82 (72–92) (*p* = 0.991); CI: baseline 3.20 (3.06–3.74) to 2.90 (2.81–3.38) (*p* = 0.173); post-induction 2.70 (2.43–3.01) to 2.70 (2.59–3.11) (*p* = 0.499); gas-insufflation 2.80 (2.44–2.98) to 2.90 (2.73–3.41) (*p* = 0.095); pfannenstiel incision 2.90 (2.78–3.25) to 2.80 (2.70–3.18) (*p* = 0.669); end of surgery 3.20 (2.79–3.33) to 3.00 (2.75–3.37) (*p* = 0.987); 24 h 3.08 (2.67–3.49) to 2.63 (2.34–2.93) (*p* = 0.071). The *p*-values were analyzed using Mann-Whitney test, *p < 0.05 is significant. QL, quadratus lumborum; MAP, mean arterial pressure

Intraoperative MAP, pulse rate and cardiac index (Fig. 4) were not significantly different between the QLB and epidural groups, however, postoperative MAP measured at 24 h after surgery was significantly lower in the epidural group 72.26 (95% CI: 67.69–76.83) mmHg than those in QLB group 83.33 (95% CI: 78.72–87.95) mmHg (*p* = 0.001) (See Additional file 3).

The Bromage scores were not significantly different between the groups. In the epidural group, only 1 subject had postoperative mild paresthesia and improving when the continuous epidural analgesia was discontinued at the end of study. The incidence of PONV in the QLB group was not significantly higher than the epidural group. Subjects in the QLB block group had a significantly shorter duration of urinary catheterization than the epidural group (Table 3). Block-related complications such as local anesthetic systemic toxicity (LAST), bleeding, infection or neurological deficits, and epidural catheter-related problems (dislodge, blocking, or leakage) were not found in this study.

**Table 3:** Postoperative side effects of QLB versus continuous epidural analgesia

Parameter	QLB group (*n* = 31)	Epidural group (*n* = 31)	*p-*value	Mean difference (95% CI)
Bromage score after anesthesia recovery				
Immediately	1 (0.96–1.12)	1 (0.96–1.12)	1.000	0 (0.11–0.11)
At 2 h	1 (0.96–1.12)	1 (0.96–1.12)	1.000	0 (− 0.11–0.11)
At 6 h	1 (0.96–1.12)	1 (1–1)	0.327	0.04 (− 0.41–0.12)
At 12 h	1 (1–1)	1 (1–1)	1.000	–
At 24 h	1 (1–1)	1 (1–1)	1.000	–
Paresthesia (%)	0 (0.00)	1 (3.22)	0.05	–
Postoperative nausea and vomiting (%)	7 (22.58)	5 (16.12)	0.07	–
Duration of urinary catheter (hours)	37.03 ± 9.14	42.97 ± 5.72	0.004	− 6.73 (− 11.25 – − 2.21)

*QLB* Quadratus lumborum block. Data are presented as mean ± standard deviation or median (95% confidence interval) or number (percentage). Numerical values are compared using unpaired t-test or and Mann-Whitney test as appropriate. Categorical values are compared using Fisher’s Exact Test-Exact sig. (1-sided) or Pearson Chi-square-Asymp. Sig. (2-sided). *P*-values of 0.05 or less are considered significant.

## Discussion

Living laparoscopic nephrectomy donors suffer more pain and are associated with more morphine consumption than other laparoscopic nephrectomy patients [14, 15]. Our study was the first randomized controlled trial comparing the analgesic efficacy of bilateral transmuscular anterior QLB with continuous thoracic epidural analgesia following transperitoneal laparoscopic living donor nephrectomy. During surgery, the intraoperative fentanyl consumption was not significantly different between the QLB and continuous epidural group. After surgery, the 24-h cumulative morphine requirement and the pain scores at rest and in movement were not significantly different between the two groups. Our results suggested that the QLB had comparable analgesic effects with continuous epidural analgesia for the first 24 h after laparoscopic nephrectomy. However, we found the pain level assessment was challenging. The PCA morphine after surgery was intended to treat surgical pain but the urinary catheter discomfort also became a trigger for the patient to use the PCA.

In our study, the dermatomal coverage of QLB extended from T10 to L2 that was lower compared to the QLB coverage from T7 to T12 in a study by Murouichi and colleagues [8]. However, the spread of local anesthetic covered the dermatome area of the Pfannenstiel incisions at T12–L1 and the analgesic effect was sufficient for surgical wound pain relief [2, 3, 16]. Although local anesthetic was injected at L4 level, the studies in cadavers and volunteers demonstrated the extensive cephalad distribution of local anesthetic to the thoracic level that may be associated with the anterolateral penetration of quadratus lumborum and anterior thoracolumbar fascia [17]. This spread has not been clearly proven, however, the QLB has the potential to provide both somatic and visceral analgesia due to the spreads of local anesthetics to the thoracolumbar fascia that was extensively innervated by the A- and C-fiber nociceptors and mechanoreceptors. The transmuscular anterior QLB (or QLB3) facilitates the spread of local anesthetic into the thoracic paravertebral space, which produces prolonged block from 6 to 48 h and achieving visceral pain relief similar to epidural block [6, 18].

As a comparison to the bilateral QLB using 0.25% bupivacaine, postoperative epidural analgesia using 6 mL/h of 0.125% bupivacaine without opioid was considered a weak analgesic treatment to achieve mild to moderate analgesia after surgery. The total dose of local anesthetic determines the quality of block and the analgesic effect that could affect our study result. A meta-analysis showed a comparable pain score between the low and high concentrations of local anesthetic, however, a higher dose may have improved and prolonged the analgesic effect [19]. The epidural block remained the effective approach to produce a reliable analgesia for abdominal surgery compared to the QLB because of the spread of local anesthetic was vary depending on the site of injection that was highly associated with the coverage and quality of analgesia [20, 21]. Therefore, the optimal dose of local anesthetic for QLB still needs to be determined.

The epidural group showed lower MAP and required more ephedrine during observation compared to the QLB group. Epidural analgesia especially using high concentration more than 0.1% bupivacaine or equivalent ropivacaine dose has unfavorable side effects such as hypotension, especially in patients at risk of hemodynamic instability [10, 19] Zhu and colleagues found the combination of thoracic epidural and general anesthesia had lower systolic, diastolic and pulse pressure compared with general anesthesia alone in laparoscopic cholecystectomy [22].

We found the QLB using 0.25% bupivacaine relieved the pain in abdominal area as effective as epidural analgesia using 0.125% bupivacaine without limb motor blockade. Both groups showed the absence of lower extremity motor blockade represented by the Bromage score (0–1). A meta-analysis summarized that 0.1% or less bupivacaine and equivalent ropivacaine epidural concentration resulted in less motor blockade, earlier ambulation, and reduced urinary retention although most of the studies analyzed were using opioid additives [19]. Paddalwar and colleagues reported the less motor block intensity without affecting the quality of analgesia in epidural analgesia using 0.125% bupivacaine [23]. The incidence of PONV was low and comparable between the QLB and epidural groups since there was no significant difference in the opioid requirement between the two groups.

Patients receiving QLB showed a significantly shorter duration of urinary catheterization than patients with continuous epidural analgesia. Early removal of the urinary catheter when it is no longer needed is recommended regarding postoperative early recovery because longer urinary catheterization can affect mobilization [24]. We noticed that some surgeons decided to remove the urinary catheter after the discontinuation of epidural analgesia due to the concern of urinary retention. From our observation, none of our patients in both groups required urinary re-catheterization, which was different from the study by Niraj and colleagues that found more patients with continuous epidural analgesia required urinary re-catheterization due to urinary retention than patients with continuous transversus abdominis plane block. Epidural analgesia acts on lumbar and sacral nerve fibers and blocks the bladder detrusor function [25]. Hayami and colleagues demonstrated that the urinary catheter removal before discontinuing epidural analgesia had higher incidences of postoperative urinary retention regardless of the amount of opioid use [25].

From the technical aspects and safety perspective, LAST was a concern in our study as the QLB was the bilateral high-volume local anesthetic block. However, the mean total bupivacaine requirement in the QLB group was significantly lower than the epidural group. We did not find any LAST symptoms during study observation given that the total dosage of local anesthetic in both groups were lower than the recommendation for bupivacaine to not exceed 2.5–3 mg/kg or 175 mg per injection with maximum dosage 400 mg in 24 h [26–28]. The thoracic epidural procedure is more challenging especially for the mid to high thoracic level compare to the lower thoracic or lumbar level. The sonoanatomic markers of the lateral (QLB1), posterior (QLB2) and intramuscular QLB are easy to find and safe to be performed in a supine position. The transmuscular anterior approach (QLB3) is considered as an invasive technique which is better performed in the lateral or supine slightly lateral position by the experienced practitioners [29].

Our study had several limitations. We did not include a control group using i.v. morphine PCA alone or the multimodal analgesia (MMA) regimen as the comparator in our study. Given that the results showed the low 24-h morphine consumption and NRS pain score, determining the true analgesic value of QLB and continuous epidural analgesia might be uncertain. However, our pain score results were lower than the pain score from a study by Gorevski and colleagues that compared i.v. PCA without any regional block to those using MMA [14]. Our results also showed the lower 24-h morphine consumptions in comparison to a study by Wang and colleagues on the laparoscopic nephrectomy patients using i.v PCA alone for the 24 h after surgery [14, 15]. Since the postoperative pain score of our study was relatively low, there was still a possibility that MMA without any regional block can achieve the same level of analgesia as the QLB or epidural analgesia alone. However, Capdevila and colleagues had proven the open nephrectomy patients receiving thoracic epidural analgesia experienced significantly less postoperative pain and morphine consumption compared to patients receiving the MMA regimen [30].

There was a lack of blinding because of the epidural block had a catheter inserted and the QLB was the bilateral injections without insertion of the catheter, but those are the common approach of the procedure in our clinical practice. The extent of block was confirmed only once after anesthesia recovery, and the analgesic efficacy was assessed only in 24 h after surgery following the policy in our institution that retains epidural catheter only for 24 h after laparoscopic surgery. Our subjects were representative of the normal BMI population that underwent laparoscopic nephrectomy in our institution. Therefore, generalizability is limited in the different population such as obese patient or other clinical contexts in which continuous QLB is available or epidural block can be maintained longer than 24 h.

## Conclusion

The QLB has potential as the alternative pain management following laparoscopic nephrectomy. The QLB had a similar 24-h cumulative morphine requirement, higher postoperative mean arterial pressure, similar postoperative pain intensity, time of first analgesics, PONV, degree of motor and sensory blockade, and shorter duration of urinary catheterization in comparison with continuous epidural analgesia after transperitoneal laparoscopic nephrectomy. Further studies of the QLB regarding the optimal dosage of local anesthetic is needed.

## Supplementary information

**Additional file 1.** Quadratus lumborum block sonography.

**Additional file 2.** Postoperative analgesic requirement of QLB versus continuous epidural analgesia.

**Additional file 3.** Perioperative hemodynamic profile of QLB versus continuous epidural analgesia.

## Data Availability

All data generated or analyzed during this study are presented in this manuscript and/or additional supporting files. The additional datasets are also available from the corresponding author on reasonable request.

## Abbreviations

ASA: American Society of Anesthesiologists classification
BMI: Body mass index
L1, L2, L3, L4: First, second, third, fourth lumbar vertebral body
LAST: Local anesthetic systemic toxicity
MAP: Mean arterial pressure
MMA: Multimodal analgesic
NRS: Numerical rating scale
PCA: Patient-controlled analgesia
PONV: Postoperative nausea and vomiting
QLB: Quadratus lumborum block
T7, T8, T9, T10, T11, T12: Seventh, eighth, ninth, tenth, eleventh, twelfth thoracic vertebral body
