# Supplementary figures and images for: A randomized controlled trial on analgesic effect of repeated Quadratus Lumborum block versus continuous epidural analgesia following laparoscopic nephrectomy
Source: BMC Anesthesiol. 2019 Dec 5;19:221. doi: 10.1186/s12871-019-0891-7 (PMC6894195; doi:10.1186/s12871-019-0891-7)

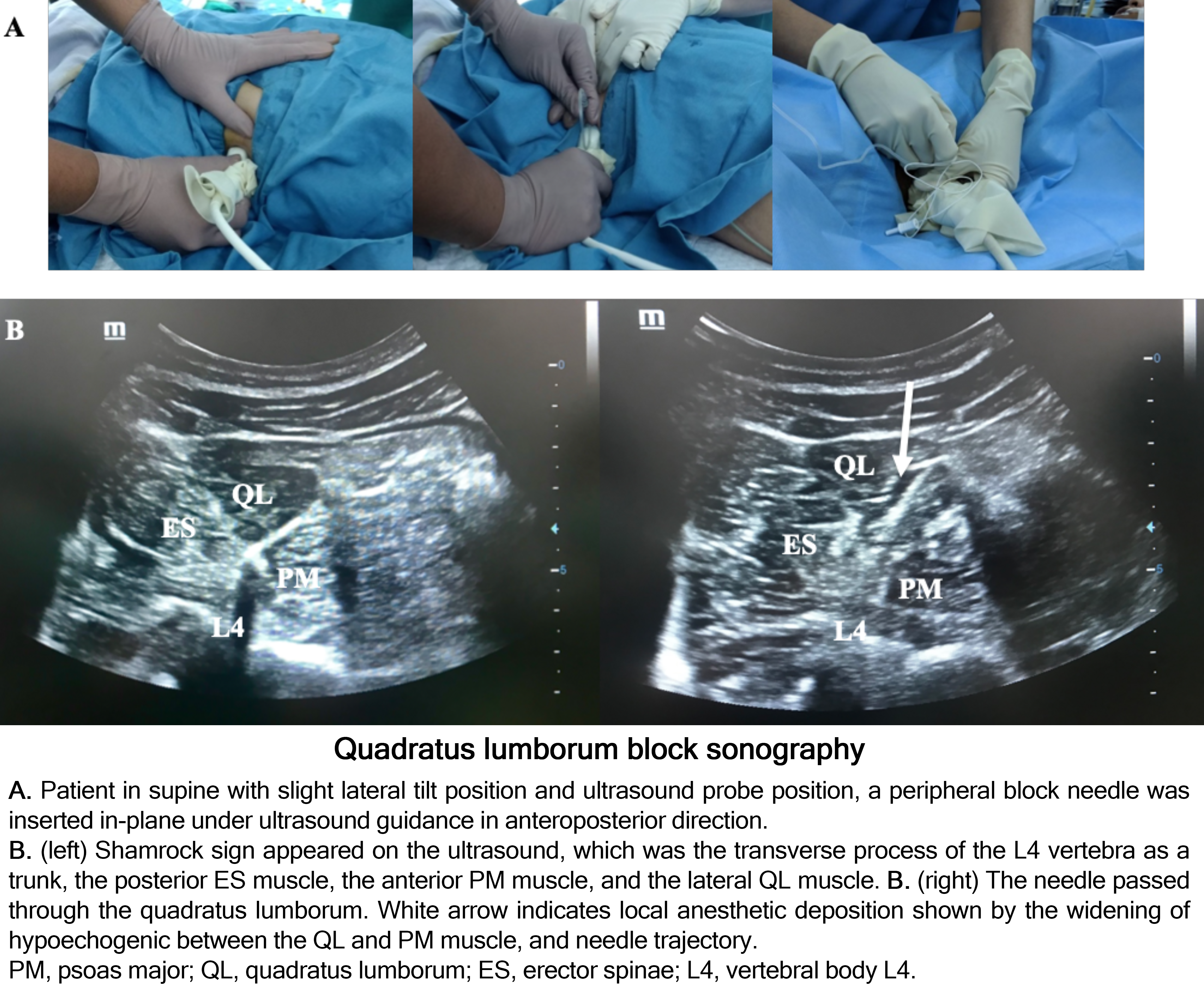

Supplement: Supplementary file 1 — Additional file 1. Quadratus lumborum block sonography. [file 12871_2019_891_MOESM1_ESM.tif]
