# Supplementary material for: A randomized controlled trial on analgesic effect of repeated Quadratus Lumborum block versus continuous epidural analgesia following laparoscopic nephrectomy
Source: BMC Anesthesiol. 2019 Dec 5;19:221. doi: 10.1186/s12871-019-0891-7 (PMC6894195; doi:10.1186/s12871-019-0891-7)
Supplement: Supplementary file 2 — Additional file 2. Postoperative analgesic requirement of QLB versus continuous epidural analgesia. [file 12871_2019_891_MOESM2_ESM.docx]

**Additional file 2.** Postoperative analgesic requirement of QLB versus continuous epidural analgesia.

| **Parameter** | **QLB group**  **(n = 31)** | **Epidural group**  **(n = 31)** | ***p* value** |
| --- | --- | --- | --- |
| **Cumulative morphine requirement after anesthesia recovery (mg)** | | | |
| At 2 hours | 0 (0.37 – 1.05) | 0 (0.33 – 1.15) | 0.857 |
| At 6 hours | 2 (1.32 – 2.94) | 2 (1.28 – 3.04) | 0.977 |
| At 12 hours | 3 (2.61 – 5.32) | 3 (2.40 – 5.34) | 0.787 |
| At 24 hours | 3 (3.63 – 7.40) | 4 (3.36 – 6.97) | 0.910 |
| **Time to first morphine requirement (minutes)** | 120 (137.61 – 354.45) | 125 (133.56 – 370.31) | 0.703 |

Statistical analysis was calculated using Mann-Whitney test. Data are presented as median (95% confidence interval), *p* < 0.05 is significant.
