# Supplementary material for: A randomized controlled trial on analgesic effect of repeated Quadratus Lumborum block versus continuous epidural analgesia following laparoscopic nephrectomy
Source: BMC Anesthesiol. 2019 Dec 5;19:221. doi: 10.1186/s12871-019-0891-7 (PMC6894195; doi:10.1186/s12871-019-0891-7)
Supplement: Supplementary file 3 — Additional file 3. Perioperative hemodynamic profile of QLB versus continuous epidural analgesia. [file 12871_2019_891_MOESM3_ESM.docx]

**Additional file 3**. Perioperative hemodynamic profile of QLB versus continuous epidural analgesia.

| **Parameter** | **QLB group**  **(n = 31)** | **Epidural group**  **(n = 31)** | ***p-*value***** |
| --- | --- | --- | --- |
| **Mean arterial pressure (mmHg)** | | | |
| Baseline | 91 (87.34–95.76) | 90 (88.36–98.99) | 0.524 |
| Post-induction | 81 (75.76–85.15) | 71 (70.36–79.06) | 0.072 |
| Gas insufflation | 92 (86.84–97.48) | 88 (82.90–92.52) | 0.210 |
| Pfannenstiel incision | 78 (75.60–84.47) | 75 (73.91–82.03) | 0.486 |
| End of surgery | 75 (72.48–81.32) | 71 (68.22–75.17) | 0.063 |
| 24 hours | 83.33 (78.72–87.95) | 72.26 (67.69–76.83) | 0.001* |
| **Pulse rate (bpm)** | | | |
| Baseline | 80 (77.67–85.30) | 88 (80.16–90.93) | 0.215 |
| Post-induction | 76 (70.87–78.10) | 73 (70.00–80.45) | 0.816 |
| Gas insufflation | 80 (71.48–81.16) | 74 (72.27–79.80) | 0.855 |
| Pfannenstiel incision | 81 (77.95–90.89) | 80 (76.75–84.73) | 0.447 |
| End of surgery | 90 (83.64–92.17) | 80 (75.14–88.02) | 0.049 |
| 24 hours | 82 (64 – 100) | 82 (72 – 92) | 0.991 |
| **Cardiac index (L/min/ m^2^)** | | | |
| Baseline | 3.20 (3.06–3.74) | 2.90 (2.81–3.38) | 0.173 |
| Post-induction | 2.70 (2.43–3.01) | 2.70 (2.59–3.11) | 0.499 |
| Gas insufflation | 2.80 (2.44–2.98) | 2.90 (2.73–3.41) | 0.095 |
| Pfannenstiel incision | 2.90 (2.78–3.25) | 2.80 (2.70–3.18) | 0.669 |
| End of surgery | 3.20 (2.79–3.33) | 3.00 (2.75–3.37) | 0.987 |
| 24 hours | 3.08 (2.67 – 3.49) | 2.63 (2.34 – 2.92) | 0.071 |

Statistical analysis was calculated using Mann-Whitney test. Data are presented as median (95% confidence interval), **p* < 0.05 is significant.
